# Investigation of Bufavirus and Parvovirus 4 in Patients with Gastro-Enteritis from the South-East of France

**DOI:** 10.3390/pathogens10091151

**Published:** 2021-09-07

**Authors:** Francis Simo-Fouda, Laurence Thirion, Antoine Nougairède, Léa Luciani, Jean-Sélim Driouich, Paul Rémi Petit, Pascal Delaunay, Remi N. Charrel

**Affiliations:** 1 Unité des Virus Émergents (UVE), Aix-Marseille Université, IRD 190-Inserm 1207, 13005 Marseille, France; francis-girauld.simo-fouda@etu.univ-amu.fr (F.S.-F.); laurence.thirion@ird.fr (L.T.); antoine.nougairede@univ-amu.fr (A.N.); lea.luciani@ap-hm.fr (L.L.); jean-selim.driouich@etu.univ-amu.fr (J.-S.D.); paul-remi.petit@etu.univ-amu.fr (P.R.P.); 2 Inserm U1065 Centre Méditerranéen de Médecine Moléculaire, Département de Parasitologie-Mycologie, Centre Hospitalier Universitaire de Nice (CHU Nice), 06202 Nice, France; delaunay.p@chu-nice.fr

**Keywords:** bufavirus, human parvovirus 4, Protoparvovirus, parvovirus, *Parvoviridae*, gastroenteritis, diarrhea, France

## Abstract

Bufavirus (BuV) and human parvovirus 4 (PARV4) belong to the *Parvoviridae* family. We assessed BuV and PARV4 DNA presence by real-time PCR analysis in stool, blood and respiratory samples collected in patients from Marseille and Nice, two large cities in the South-East of France. Bu-V DNA was detected in diarrheic stool samples from 92 patients (3.6% of 2583 patients), particularly men and adults, and patients from the nephrology and the infectious disease departments. Among the patients with a BuV-positive stool sample and for whom at least one blood sample was available (*n* = 30 patients), BuV DNA was detected also in 3 blood samples. In contrast, BuV DNA was not detected in any of the respiratory samples from 23 patients with BuV-positive stool. BuV detection rate was comparable in stool samples from patients with and without diarrhea. We did not detect PARV4 DNA in any of the stool specimens (*n* = 2583 patients). Our results suggest that PARV4 fecal–oral transmission is rare or non-existent in the South-East of France while BuV circulates with a relatively high rate in this area.

## 1. Introduction

Viruses of the *Parvoviridae* family are small single-stranded DNA viruses that infect a wide range of organisms, from invertebrates to humans. The first human parvovirus was discovered in 1965 and since 2005, many new parvoviruses have been identified in human samples [1,2].

Bufavirus (BuV), a virus from the genus *Protoparvovirus*, was identified in 2012 from fecal samples of children with diarrhea in Burkina Faso [3,4]. To date, three genotypes, sharing 65–73% amino acid identity within VP2, have been described [1,5]. BuV DNA has been detected in stool samples (prevalence ranging from 0 to 4%), mostly from patients presenting with diarrhea, in many countries. Other populations have been rarely studied and a limited number of patients has been tested: BuV was not found in blood or cerebrospinal fluids (*n* = 126) in Turkey; Buv DNA was detected in 1/995 in nasal swabs in Finland and 0/44 nasopharyngeal aspirate from Latvia; studies conducted in Finland, Turkey and Thailand detected BuV DNA in diarrheic samples but not in non-diarrheic samples, whereas studies in Bangladesh and Malawi reported BuV DNA in both diarrheic and non-diarrheic samples [6,7,8,9,10,11,12]. The causative role of BuV in gastroenteritis remains unclear, and its association with other clinical presentations remains largely to be explored [5,6,8]. 

Human parvovirus 4 (PARV4), a virus belonging to the genus *Tetraparvovirus* was first described in 2005 in plasma from patients with acute viral infection syndrome. It has been classified in three genotypes, and genotype 2 was formerly known as PARV5 [5,13,14]. The low genetic diversity between PARV4 genotypes (<3% at the amino acid level) supports that introduction in human populations is likely to have occurred recently [15]. PARV4 has been detected in blood/plasma samples, autopsy samples, stools, nasopharyngeal swabs, bone marrow and cerebrospinal fluid; the clinical impact of PARV4 infection remains uncertain, but reported disease associations include influenza-like syndrome, encephalitis, worsening of HIV evolution and fetal hydrops; PARV4 association with gastroenteritis remains to be established [5,13,15,16].

The aims of the present study were (i) to investigate BuV and PARV4 presence in stool samples from patients with diarrhea in the South-East of France; (ii) to check whether in patients with positive stools, both viruses can be found also in blood and respiratory samples; (iii) to determine whether the detection rate of these viruses varies in patients with and without diarrhea in hospital settings.

## 2. Results and Discussion

### 2.1. BuV and PARV4 Detection in Stool Samples from Patients with Diarrhea (Cohort 1)

#### 2.1.1. Parvovirus 4 Detection

None of the 3148 stool samples of cohort 1 contained PARV4 DNA. Although PARV4 has been detected in stool samples in Ghana (0.53%, 5/943 fecal samples from children) [17], our results suggest that PARV4 fecal–oral transmission is rare or non-existent in the South-East of France.

#### 2.1.2. Bufavirus Detection

##### Demographic Data

The median age of the 2583 patients of cohort 1 was 56 years (range 0 to 101 years) and 1305 (50.5%) were men (Table 1).

Samples (stool, blood and respiratory specimens) were tested for the presence of BuV DNA using a real-time quantitative PCR assay described by one of the leading research group in the field [18]. In total, BuV DNA was detected in 3.6% of patients (*n* = 92/2583, *n* = 107 stool samples) of cohort 1 (Table 1). This positivity rate is similar to the one reported by Smits et al. in the Netherlands (3.7%) [19], but it is higher than observed in Finland (1.1%) [18], China (1.7%) [20] and Thailand (0.3%) [10]. The difference in BuV detection rate could be affected by method used for samples testing, as we used real-time PCR with positivity cutoff at a cycle threshold of <40, while in Netherland authors used viral metagenomics approach, or nested PCR in Thailand; in Finland and China authors used real-time PCR without detailing the cut-off value [10,18,19,20]. Moreover, a study by the French Reference Center for gastroenteritis viruses reported a detection rate of 15.8% (*n* = 85/538 stool samples) in children admitted to the Dijon and Saint-Etienne University Hospital Centers in 2019 [21]. If confirmed, this and our results suggest that BuV is circulating at higher rate in France than in other countries.

The age of BuV-positive patients ranged from 1 to 92 years (median: 58 years). BuV detection rate in >15-year-old patients (4.3%, 88/2044) was significantly higher than in ≤15-year-old patients (0.7%, 4/539, *p* < 0.0001) (Table 1). Similar epidemiological trends were reported in Finland, in the Netherlands, in China and in Thailand. For instance, in Thailand, the BuV DNA detection rate in children was 0.07% (one case) compared with 3.7% in adults [10].

The men/women ratio was 3 in the BuV-positive group, and BuV detection rate was significantly higher in men than women (5.3%, 69/1305, vs. 1.8%, 23/1278; *p* < 0.0001), as observed in Finland (sex ratio of 2.5) [18]. In other studies, the sex differences in detection rate were not significant [10,19,20].

Our results did not suggest that BuV is a seasonal virus (Table 1), in agreement with previous data [18]. In contrast, temporal clustering associated with the cold season was observed in China and in Turkey [11,20].

##### Characteristics of BuV-Positive Patients

The BuV detection rate was higher in patients from the Nephrology (9.3%, 19/204 stool samples from 18 patients) and Infectious Disease (6.0%, 13/215 stool samples from 12 patients) departments compared with other departments (detection rates < 5%).

**Figure 1:**
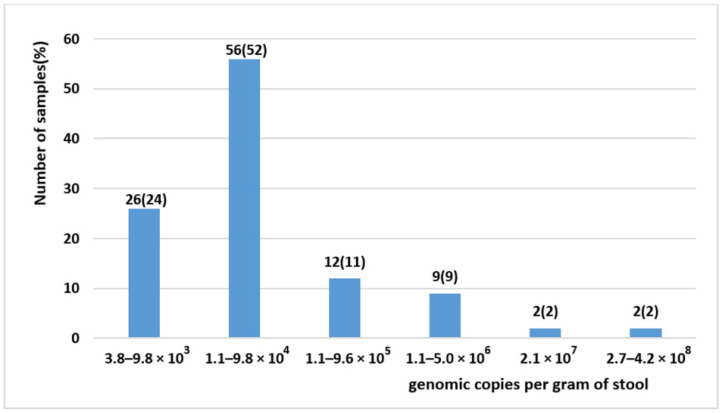
BuV viral load in the 107 BuV-positive stool samples.

The viral load was determined in the 107 BuV-positive stool samples collected from 92 patients and ranged from 3.8 × 10^3^ to 4.2 × 10^8^ genome copies per gram of stool (or per mL of 15% fecal suspension), with mean and median values of 7.1 × 10^6^ and 2.8 × 10^4^ genome copies per gram of stool (Figure 1). Previous studies reported values that were lower in stool samples [9,18,19], with values ranging between 2.1 × 10^2^–1.6 × 10^3^ [9] and 1.9 × 10^3^–3.2 × 10^4^ [18] copies/mL of 10% fecal suspension. In addition, 23.4% (25/107) of our samples had a viral load ≥1.1 × 10^5^ genome copies per gram of stool. Previous studies showed other viruses causing gastroenteritis displayed similar viral loads. For instance, a report on children with gastroenteritis found that the adenovirus median viral load was 4.56 × 10^3^ genome copies per gram of stools (range 2.14 × 10^2^–1.32 × 10^5^) [22]. Another study on patients with gastroenteritis showed that norovirus G1 median viral load was 8.4 × 10^5^ genome copies per gram of stools (range: 2.2 × 10^4^–2.9 × 10^10^) [23]. These data suggest that BuV might be a diarrhea-causing virus in some patients who should be identified in specific studies.

BuV could lead to chronic infection or carriage. Among the 11 patients with available sequential stool samples, five (#1, #14, #19, #38 and #82) (Table 2) had BuV-positive samples for a period ≥3 weeks (26, 27, 49, 62 and 245 days, respectively). 

**Figure 2:**
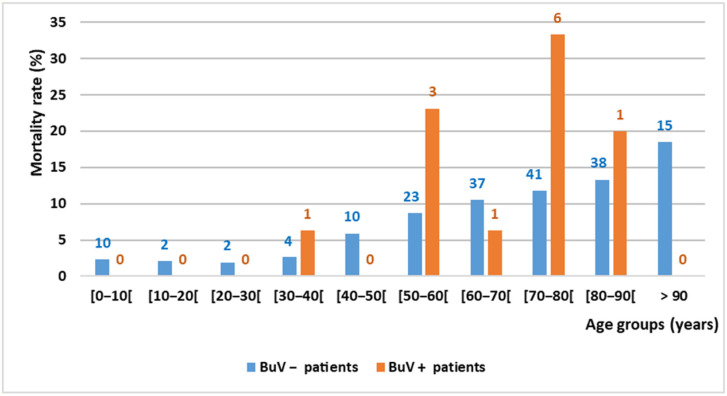
Mortality rate by age group and BuV DNA status in Marseille (3 months after stool sample collection). The number above each column indicates the number of deceased patients.

Three months after the stool samples were collected, the death rate was significantly higher among BuV-positive than BuV-negative patients (14.5%, 12/83, vs. 7.9%; 182/2292; *p* = 0.034). One year later, the death rate was still higher among BuV-positive than BuV-negative patients (16.9%, 14/83, vs. 10.8%, 247/2292), but this difference was not significant (*p* = 0.10). The causes of death of these 14 BuV-positive patients were cancer (#21, #29, #32, #41, #74, #77 and #82), pneumopathy (1#, #46, 49#, 56# and 85#), mediastinitis (#14) and vascular infection complication (#91). Comorbidities, particularly kidney failure (7/14), were recorded for 11 of these 14 patients. The mortality rates according to the age class are shown in Figure 2.

BuV has been discovered recently, and data on whether it can cause other diseases or syndrome are limited [1]. Indeed, this is the first time that data from >10 BuV-positive patients are used to investigate BuV infection. We do not know why the death rate was higher in BuV-positive than in BuV-negative patients (14.5% vs. 7.9%), and if confirmed, should be thoroughly investigated. Data on fatal outcome among BuV-positive patients are scarce. Chieochansin et al. reported that among four BuV-positive patients in Thailand one 90-year-old patient died due to congestive heart failure [10]. This underlines the need of additional studies. As 7/14 of deceased patients in our study had cancer, the link between BuV and cancer also should be explored. However, the possibility that BuV acquisition or prolonged shedding may be facilitated by the failing conditions encountered in immunocompromised patients should also be considered. Interestingly, cutavirus, a close BuV relative, has been detected in skin biopsies of patients with cutaneous lymphoma (4/17, France) and malignant skin lesions (1/10, Denmark). Moreover, such as BuV, it has been found in stool samples from patients with diarrhea (4/245 Brazil; 1/100 Botswana) [24,25,26].

##### BuV Co-Infection

Among the 107 BuV-positive stool samples, adenovirus was co-detected in 7 samples (6 patients), norovirus GII in 3 samples (2 patients), norovirus GI and astrovirus in 1 sample/each. BuV, adenovirus and norovirus GII were simultaneously detected in the stool sample of a HIV-positive patient. Conversely, rotavirus and sapovirus were not detected in the BuV-positive stool samples. Several previous studies reported co-infection of BuV with norovirus, although co-infection with astrovirus was the first to be reported [9,11,18,20,27,28]. Co-infection of bufavirus and adenovirus could be explored once we know that members of the *Dependoparvovirus* genus in the same family compensate for their genetic limitations by co-opting helper viruses, most commonly adenoviruses or herpesviruses [5].

### 2.2. BuV Testing in Blood and Respiratory Samples from Patients with Buv-Positive Stool Samples

Among the 14 patients with BuV-positive stool samples who died, blood samples were available for 6 of them (14 samples). BuV DNA was detected in blood samples of two of these patients, with a viral load ranging from 2.8 × 10^3^ to 2.0 × 10^4^ genome copies/mL of blood (Table 2). In BuV-positive patients with available blood samples, BuV was more frequently detected in blood samples of patients who died (2/6 patients vs. 1/24 patients) (Table 2). In Switzerland, BuV nucleic acids were detected in 1/25 plasma samples; this patient presented with acute leukemia who underwent bone marrow transplantation and the issue was fatal 2 months later; the same patient had also a BuV-positive stool sample [29]. Together with our results, this supports that BuV could be involved in extra-intestinal clinical manifestations. Last, specific antibodies were detected in 13.2% of patients in another region of France [30], and in 1.9%, 3.6%, 56.1%, 72.3% and 84.8% of patients from cohorts from Finland, USA, Iran, Kenya and Iraq, respectively [2].

Finally, 27 oral and respiratory samples (8 saliva, 6 pleural fluids, 6 bronchoalveolar lavages, 4 nasopharyngeal aspirations and 3 sputum) were available for 23 of the 83 BuV-positive patients from Marseille. All these samples were BuV-negative by PCR, in agreement with results in Finland where only one of 955 nasal swab specimens was positive [9]. Whether BuV can cause respiratory infection can clearly be questioned and justifies further studies designed specifically to lift the veil.

### 2.3. BuV Detection Rate in Stool Samples from Patients with and without Diarrhea (Cohort 2)

In cohort 2 (30–75-years-old patients), BuV DNA positivity was not significantly different in patients with and without diarrhea: 10.50% vs. 10.00% (*p-value = 1*) for men, and 4.59% vs. 4.41% (*p-value = 1*) for women (Table 3). Among the BuV-positive samples, only one from the group with diarrhea, was also positive for another pathogen (*Tropheryma whipplei*). 

Data on BuV DNA detection in adult populations without diarrhea are scarce. In Thailand, 15 adults without diarrhea were all BuV-negative, whereas 3/81 adult patients with diarrhea were BuV-positive [10]. BuV DNA was detected in 1/227 children without diarrhea in Bangladesh [12], and in a cohort of 164 children from Malawi where the unique BuV DNA-positive sample belonged to the group without diarrhea [6].

Viral loads were higher in stool samples from the group with diarrhea than without diarrhea in cohort 2 (Tables S1 and S2 of supplemental data).

BuV detection rates in 30–75-year-old men and women of cohort 2 were higher than those obtained for cohort 1 with respectively detection rates of 10.5% vs. 7.4% (*p-value = 0.2*) in men and 4.6% vs. 3.0% (*p-value = 0.5*) in women (Table 4). This could reflect that BuV circulation might be increasing in Marseille from 2017 to 2021 although the lack of comparability between the two cohort might have introduced a bias. This merit to be confirmed in future studies.

### 2.4. Genotyping of BuV-Positive Samples

Genotyping has been attempted for the 92 patients, which had positive results for BuV detection (Table 2). A total of 54 were identified as BuV-1, 3 as BuV-2 and 11 as BuV-3: so, a total of 68/92 (74%) of patients were infected with a single genotype. Another 14 patients showed co-infection with two or three genotypes detected in the same clinical sample; 10 patients were negative for the three genotypes (despite BuV-positive result) and remain to be investigated further.

In conclusion, our study shows that (i) PARV4 fecal–oral transmission is rare or non-existent in the South-East of France; (ii) BuV circulates with a relatively high rate in the South-East of France; (iii) BuV circulation is not associated with seasonality; (iv) adults are more frequently infected than children, and men are more frequently infected than women; (v) BuV might lead to chronic infection or carriage in patients without diarrhea; (vi) genotype 1 is largely dominant in south-eastern France. 

## 3. Materials and Methods

### 3.1. Study Population

Two cohorts were obtained from Marseille and/or Nice.

Cohort 1 included (i) 2375 patients (*n* = 2874 diarrheic stool samples collected between March 2017 and February 2019) from different departments of Marseille University Hospital Center (Emergency, Pediatrics, Internal Medicine, Infectious Diseases, Nephrology, Gastroenterology, Surgery, Hematology and Immunology, Oncology, Cardiovascular Medicine and Geriatrics); (ii) 208 patients (*n* = 274 diarrheic stool samples collected between January 2017 and March 2018) from the Parasitology Department of Nice University Hospital Center. From patients with BuV-positive stool samples in Marseille, blood and respiratory samples were searched for availability in the Biobank; when available. Selection criteria were as follows: blood and respiratory samples were searched approximately 1 month before or after the BuV-positive stool sample was collected.

Cohort 2 included 30–75-year-old patients from different clinical departments of Marseille University Hospital Center. This cohort consisted of 2 groups: the patients with diarrhea (328 patients including 219 men and 109 women, 395 stool samples) and patients without diarrhea (246 patients including 110 men and 136 women, 261 stool samples). Stool samples were collected between November 2020 and April 2021. The hospital records of the 246 patients without diarrhea indicated that 260/261 stool samples were tested mostly for *T. whipplei* (9/153 positive samples by PCR), and that 5/86 stool samples were positive for other bacteria by culture or PCR (1 *Clostridium difficile*), 1/30 was positive for *Blastocystis hominis* and 0/8 were positive for viruses. A similar search for the group with diarrhea allowed obtaining data for 393/395 stool samples: 5/128 were positive for *T. whipplei*, 27/297 were positive for other bacteria by culture or PCR (14 *C. difficile*), 2/37 were respectively positive for *Giardia intestinalis + Blastocystis hominis* and *Oestrus ovis* and 2/54 were, respectively, positive for Norovirus GI and Cytomegalovirus.

### 3.2. Sample Preparation

For stool collection, a pea size stool sample (around 33 mg) or 300 µL of liquid stools was taken using an inoculation loop or micropipette. It was then put in a sterile tube containing 1ml of distilled water and mixed by vortexing before storage at −20 °C until use.

After thawing and mixing, approximately 10 µL of each stool sample was transferred with an inoculation loop to a conical Falcon tube containing 2 mL of distilled water and mixed by vortexing. Then 200 µL of this suspension was transferred into a S-Block (Qiagen^®^, Venlo, The Netherlands) for nucleic acid extraction with Qiacube (Qiagen^®^).

Blood and respiratory samples were collected by clinicians and kept at −80 °C until use. Frozen blood samples were thawed and mixed, and then 100 µL of blood sample was diluted in 100 µL of 0.9% NaCl, and 200 µL of this dilution was transferred into a S-Block (Qiagen^®^) for nucleic acid extraction with Qiacube (Qiagen^®^). Frozen respiratory samples were thawed and mixed, and then 200 µL of each sample was transferred into a S-Block (Qiagen^®^;) for nucleic acid extraction with Qiacube (Qiagen^®^).

### 3.3. Nucleic acid Extraction

DNA was isolated from stool, blood and respiratory samples with the Cador^®^ Pathogen 96 Qiacube HT Kit (Qiagen^®^) and the Qiacube HT device (Qiagen^®^) following the manufacturer’s recommendations with some modifications. For a 96-well plate, VXL lysis buffer included 17 mL of VXL buffer, 2.2 mL of proteinase K, 100 µL of RNA carrier (1 µg/µL) and 1 mL of bacteriophages MS2/T4 as internal control. The AVE Buffer elution volume was 150 μL per well for stool samples, and 90 µL for blood and respiratory samples.

### 3.4. Sample Testing

By using primers and hydrolysis probes targeting the NS1 region of the virus, BuV presence was tested in all stool samples (cohort 1 and 2) as previously described [18]. The presence of PARV4, genotypes 1 and 2, and PARV4 genotype 3 was tested only in stool samples from cohort 1 by using previously described primers and hydrolysis probes specific to ORF2 region for PARV4 genotypes 1 and 2 [31], and NS1 region for PARV4 genotype 3 [32]. BuV-positive stool samples from cohort 1 were also tested to determine the presence of norovirus GI [33], norovirus GII [34], rotavirus (with the Rota 2 Fwd ACCATCTWCACRTRACCCTCTATGAG, Rota 2 Rev GGTCACATAACGCCCCTATAGC and Rota 2 P FAM-AGTTAAAAGCTAACACTGTCAA-BHQ primers adapted from Freeman M et al., 2008) [35], sapovirus [36], astrovirus [36] and adenovirus [37]. BuV presence was also tested in the available blood and respiratory samples of patients with BuV-positive stool samples (cohort 1). Data from the second cohort have been dedicated to section on BuV detection rate in stool samples from patients with and without diarrhea; all others results sections are based on data of the first cohort. 

### 3.5. Positive Control

In-house designed plasmids were used as positive controls for BuV, PARV4 genotypes 1 and 2, and PARV4 genotype 3. These plasmids included the BuV/PARV4 DNA sequences targeted by the primers/probes used for pathogen detection, the promoter T7, and *BamH*I, *Xma*I and *Not*I restriction sites.

### 3.6. Taqman Real-Time PCR Assays 

Real-time PCR was performed on a Bio-Rad CFX96^TM^ Real-Time System, software version 3.1 (Bio-Rad Laboratories, Hercules, CA, USA), using LightCycler^®^ 480 Probes Master (Roche Diagnostics, Germany) and a total volume of 30 μL (10 μL of DNA and 20 μL of reaction mix). Each reaction mix contained 2.6 µL of PCR-grade water, 15 µL of LightCycler 480 Probes Master 2×, 1 µL (333.3 nM) of forward and reverse primers, and 0.4 µL (133.3 nM) of probe. The thermal cycle program was: 10min at 95 °C, followed by 45 cycles of 10sec at 95 °C, 30 s at 60 °C and 1 s at 72 °C. Samples were classified as positive at a cycle threshold of <40. 

Genotyping was performed using 3 specific real-time quantitative PCR assays each targeting one genotype (detailed in Supplementary Table S3).

For quantitation, serial 10-fold dilutions of the plasmid (2.88 × 10^11^ copies/µL) containing the BuV NS1 insert targeted by primers and probes were used to calibrate the Bio-Rad CFX96^TM^ Real-Time System. The resulting linear equation and the mean weight of stool samples were used to estimate the viral load with Microsoft Excel 2016. 

### 3.7. Statistical Analysis 

For data analysis, the Fisher’s exact test was used to compare percentages with the SPSS statistical package release 17.0 (SPSS Inc., Chicago, IL, USA).

## Figures and Tables

**Table 1:** Socio-demographic characteristics of the patients in cohort 1.

	BuV DNA + Patients (*N* = 92)	*p-Value*	Study Population (*N* = 2583)
**Residence, *n* (%)**			
Marseille	83 (3.5)		2375
Nice	9 (4.3)		208
**Sex, *n* (%)**			
Men	69 (5.3)		1305
Women	23 (1.8)		1278
Sex Ratio (M/W)	3.00	*<0.0001*	1.02
**Age, Years**			
Median	58		56
Range	1–92		0–101
Children (≤15 years)	4 (0.7)		539
Adults (>15 years)	88 (4.3)		2044
		*<0.0001*	
**Age/Sex Groups, *n* (%)**			
Young (≤15-Year-old) Men	4 (1.4)		286
Young (≤15-Year-old) Women	0 (0.0)		253
Adult (>15-Year-old) Men	65 (6.4)		1019
Adult (>15-Year-old) Women	23 (2.2)		1025
		*<0.0001*	
**Seasonality; *n* (%)**			
Cumulated Winters	36 (3.17)		1136
Cumulated Springs	26 (3.45)		754
Cumulated Summers	14 (3.33)		420
Cumulated Autumns	31 (3.70)		838
		*0.94*	

**Table 2:** BuV viral load in stool samples and blood samples (cohort 1).

N.	Sex	N of Days *	BuV Viral Load in Stool Samples **	Others Viruses Found in Stool	Blood Samples *** (Days before Death)
***1***	***M***	***1***	***−***	***−***	***Negative (64)***
		***9***	***4.2 × 10^8^***	***ADV ^†^ and Noro ^‡^ GII***	***−***
		***57***	***−***	***−***	***2.0 × 10^4^ (8)***
		***58***	***2.7 × 10^8^***	***ADV and Noro GII***	***−***
		***61***	***−***	***−***	***3.3 × 10^3^ (4)***
		***64***	***−***	***−***	***5.8 × 10^3^ (1)***
2	M	*−*	2.1 × 10^7^	*−*	*−*
3	M	*−*	2.1 × 10^7^	*−*	*−*
4	M	1	5.0 × 10^6^	ADV	Negative
5	F	−	4.6 × 10^6^	−	−
6	F	−	2.7 × 10^6^	−	−
7	M	−	2.4 × 10^6^	−	−
8	M	−	1.7 × 10^6^	−	−
9	M	1	1.7 × 10^6^	−	−
		10	6.8 × 10^5^	−	−
		14	−	−	Negative
10	M	−	1.6 × 10^6^	−	−
11	F	−	1.1 × 10^6^	−	−
12	M	−	9.6 × 10^5^	−	−
13	M	1	9.1 × 10^5^	−	Negative
***14***	***M***	***1***	***−***	***−***	***Negative (82)***
		***5***	***8.6 × 10^5^***	***−***	***−***
		***22***	***−***	***−***	***Negative (61)***
		***32***	***2.8 × 10^4^***	***−***	***−***
15	M	1	7.9 × 10^5^	−	−
		8	−	−	Negative
16	M	1	−	−	Negative
		3	4.6 × 10^5^	−	−
17	M	−	2.7 × 10^5^	−	−
18	F	−	2.3 × 10^5^	−	−
**19**	**F**	**1**	**1.7 × 10^5^**	**−**	**−**
		**189**	**4.7 × 10^4^**	**−**	**−**
		**189**	**3.0 × 10^4^**	**−**	**−**
		**245**	**1.1 × 10^6^**	**−**	**−**
20	M	1	1.6 × 10^5^	−	−
		1	3.8 × 10^4^	−	−
*21*	*M*	*−*	*1.3 × 10^5^*	*−*	*−*
22	F	−	9.8 × 10^4^	ADV	−
23	M	−	8.6 × 10^4^	−	−
24	M	1	8.4 × 10^4^	−	−
		6	3.0 × 10^4^	−	−
25	F	−	8.3 × 10^4^	−	−
26	M	−	6.3 × 10^4^	−	−
27	M	−	5.6 × 10^4^	ADV	−
28	M	−	5.4 × 10^4^	−	−
*29*	*M*	*−*	*5.3 × 10^4^*	−	−
30	F	−	5.3 × 10^4^	−	−
31	M	−	5.2 × 10^4^	−	−
*32*	*M*	*1*	*5.0 × 10^4^*	−	−
		*215*	*−*	−	*Negative (16)*
33	F	−	4.9 × 10^4^	−	−
34	M	−	4.7 × 10^4^	−	−
35	M	1	−	−	Negative
		5	4.6 × 10^4^	−	−
36	M	−	4.5 × 10^4^	−	−
37	M	1	4.1 × 10^4^	−	−
		2	−	−	Negative
		67	Negative	−	
**38**	**M**	**1**	**3.9 × 10^4^**	**−**	**−**
		**26**	**7.1 × 10^3^**	**−**	**−**
39	M	1	3.8 × 10^4^	−	Negative
40	F	1	−	−	Negative
		2	3.3 × 10^4^	−	−
*41*	*F*	*−*	*3.2 × 10^4^*	−	−
42	M	1	3.1 × 10^4^	−	Negative
43	F	−	3.0 × 10^4^	−	−
44	M	1	−	−	1.2 × 10^3^
		4	3.0 × 10^4^	−	−
		9	2.3 × 10^4^	−	
45	F	−	2.9 × 10^4^	−	−
*46*	*M*	*−*	*2.8 × 10^4^*	−	−
47	M	−	2.7 × 10^4^	−	−
48	F	−	2.6 × 10^4^	−	−
*49*	*M*	*−*	*2.3 × 10^4^*	−	−
50	M	−	2.2 × 10^4^	ADV	−
51	M	1	2.2 × 10^4^	ADV	−
		7	−	−	Negative
52	F	1	−	−	Negative
		5	2.0 × 10^4^	−	−
		5	1.7 × 10^4^	−	−
53	M	−	1.9 × 10^4^	−	−
54	M	1	1.7 × 10^4^	−	−
		7	1.8 × 10^4^	−	−
55	M	1	−	−	Negative
		4	1.7 × 10^4^	−	−
*56*	*M*	*−*	*1.5 × 10^4^*	−	−
57	M	−	1.5 × 10^4^	−	−
58	F	1	1.4 × 10^4^	−	−
		417	Negative	−	−
59	M	1	1.4 × 10^4^	−	Negative
60	F	−	1.4 × 10^4^	−	−
61	F	−	1.3 × 10^4^	−	−
62	M	1	−	−	Negative
		2	1.3 × 10^4^	−	−
63	M	−	1.3 × 10^4^	−	−
64	F	1	1.2 × 10^4^	−	−
		6	1.9 × 10^4^	−	−
65	M	1	−	−	Negative
		16	1.2 × 10^4^	−	−
66	M	−	1.2 × 10^4^	−	−
67	F	−	1.1 × 10^4^	−	−
68	M	−	9.8 × 10^3^	−	−
69	M	1	9.8 × 10^3^	Norovirus GII	Negative
70	F	−	9.3 × 10^3^	−	−
71	M	−	9.2 × 10^3^	−	−
72	M	1	8.7 × 10^3^	−	−
		3	−	−	Negative
73	M	1	8.2 × 10^3^	Norovirus GI	Negative
*74*	*M*	*−*	*8.1 × 10^3^*	−	−
75	M	1	−	−	Negative
		12	8.1 × 10^3^	−	−
76	M	−	7.9 × 10^3^	Astrovirus	−
*77*	*M*	*1*	*−*	*−*	*Negative (41)*
		*5*	*7.7 × 10^3^*	*−*	*Negative (37)*
		*9*	*Negative*	*−*	*−*
		*38*	*−*	*−*	*Negative (4)*
78	M	1	−	−	Negative
		2	Negative	−	−
		3	7.6 × 10^3^	−	−
79	M	1	7.1 × 10^3^	−	−
		14	1.1 × 10^4^	−	−
80	M	1	6.5 × 10^3^	−	Negative
81	M	−	6.5 × 10^3^	−	−
***82***	***M***	***1***	***Negative***	***−***	
		***215***	***6.5 × 10^3^***	***−***	***Negative (83)***
		***277***	***1.1 × 10^5^***	***−***	***−***
		***284***	***−***	***−***	***2.8 × 10^3^ (15)***
83	F	−	6.4 × 10^3^	−	−
84	F	−	6.2 × 10^3^	−	−
*85*	*M*	*1*	*−*	*−*	*Negative (76)*
		*3*	*−*	*−*	*Negative (74)*
		*77*	*6.1 × 10^3^*	*−*	*−*
86	M	1	5.6 × 10^3^	−	−
		4	Negative	−	
87	M	−	5.6 × 10^3^	−	−
88	M	−	5.4 × 10^3^	−	−
89	M	1	−	−	Negative
		3	5.1 × 10^3^	−	−
90	M	−	4.7 × 10^3^	−	−
*91*	*M*	*−*	*4.1 × 10^3^*	−	−
92	M	−	3.8 × 10^3^	−	−

M = male; F = female; * = number of days after collection of the first sample (day 1; not indicated when only one sample was collected); ** = genome copies per gram of stool; *** = genome copies per milliliter of blood; ^†^ ADV = adenovirus; ^‡^ Noro = norovirus; *in italic* = deceased patients; in bold = sequential stool samples positives for a period ≥ 3 weeks.

**Table 3:** BuV DNA detection rate in stool samples of adult patients with and without diarrhea, collected from November 2020 to April 2021 (Marseille).

		BuV + Patients	Study Population (30–75 Years of Age)
	Men	23 (10.50)	219
Patients with Diarrhea; *n* (%)	Women	5 (4.59)	109
*N* = 328	Men/Women Ratio	23/5 (4.60)	219/109 (2.01)
	Age, Range (Median), Years	30–75 (60)	30–75 (61)
	Men	11 (10.00)	110
Patients without Diarrhea; *n* (%)	Women	6 (4.41)	136
*N* = 246	Men/Women Ratio	11/6 (1.83)	110/136 (0.81)
	Age, Range (Median), Years	33–74 (61)	30–75 (53)

**Table 4:** BuV detection rate in 30−75-year-old patients with diarrhea from cohort 1 and cohort 2 (Marseille).

		BuV + Patients	Study Population (30–75 Years of Age)
Cohort 1, November 2017–April 2018; *n* (%) *N* = 578	Men	23 (7.4)	311
	Women	8 (3.0)	267
	Men/Women ratio	23/8 (2.9)	311/267 (1.2)
	Age, range (median), years	30−75 (59)	30−75 (60)
Cohort 2, November 2020–April 2021; *n* (%) *N* = 328	Men	23 (10.5)	219
	Women	5 (4.6)	109
	Men/Women ratio	23/5 (4.6)	219/109 (2.0)
	Age, range (median), years	30–75 (60)	30–75 (61)

## Data Availability

All data are provided in the article.

## Supplementary Materials

The following are available online at https://www.mdpi.com/article/10.3390/pathogens10091151/s1. Table S1. BuV viral load in diarrheic stools samples (cohort 2). Table S2. BuV viral load in non-diarrheic stools samples (cohort 2). Table S3. Real-time PCR assay used for genotyping BuV-DNA positive samples.
