# Supplementary material for: Investigation of Bufavirus and Parvovirus 4 in Patients with Gastro-Enteritis from the South-East of France
Source: Pathogens. 2021 Sep 7;10(9):1151. doi: 10.3390/pathogens10091151 (PMC8472301; doi:10.3390/pathogens10091151)
Supplement: Supplementary file 1 [file pathogens-10-01151-s001.zip › pathogens-1318115-supplementary.pdf]

Table S1. BuV viral load in diarrheic stools samples (cohort 2).

N.	Sex*	Age	BuV viral load**	BuV viral load*** (Liquid samples)	Sampling month
1	M	44	4.96×10^3	–	Nov. 2020
2	M	55	8.78×10^3	–	Apr. 2021
3	M	49	9.61×10^3	–	Dec. 2020
4	M	74	9.86×10^3	–	Mar. 2021
5	F	56	1.01×10^4	–	Feb. 2021
6	M	46	1.06×10^4	–	Apr. 2021
7	F	70	1.16×10^4	–	Nov. 2020
8	M	50	1.18×10^4	–	Nov. 2020
9	M	61	1.27×10^4	–	Dec. 2020
10	M	69	1.28×10^4	–	Nov. 2020
11	M	34	(Day1) 1.92×10^4<br>(Day5) 1.39×10^4	–<br>–	Apr. 2021
12	F	51	2.34×10^4	–	Mar. 2021
13	M	33	2.34×10^4	–	Apr. 2021
14	M	30	(Day 1) 2.77×10^4<br>(Day 5) 4.81×10^4	–<br>–	Feb. 2021
15	M	74	3.27×10^4	–	Feb. 2021
16	F	49	(Day1) 4.87×10^4<br>(Day1) 8.24×10^4	–<br>–	Jan. 2021
17	M	71	6.63×10^4	–	Dec. 2020
18	M	66	(Day 1)<br>8.09×10^4	(Day 9) 1.35×10^7	Dec. 2020
19	M	72	8.30×10^4	–	Nov. 2020
20	M	60	1.44×10^5	–	Apr. 2021
21	M	68	3.92×10^5	–	Dec. 2020
22	M	70	(Day 7)<br>4.57×10^5	(Day 1) 2.95×10^7	Jan. 2021
23	M	57	–	1.41×10^6	Feb. 2021
24	F	60	–	1.47×10^6	Mar. 2021
25	M	67	–	1.60×10^6	Jan. 2021
26	M	63	–	1.67×10^6	Nov. 2020
27	M	46	–	2.13×10^6	Feb. 2021
28	M	75	–	2.99×10^6	Dec. 2020

*M = male; F = female; ** = genome copies per gram of stool; *** = genome copies per mL of stool.

Table S2. BuV viral load in non-diarrheic stools samples (cohort 2).

N.	Sex*	Age	BuV Viral Load**	Sampling Month
1	F	53	8.03×10^3	Nov. 2020
2	M	71	8.78×10^3	Feb. 2021
3	M	62	9.31×10^3	Mar. 2021
4	M	55	9.37×10^3	Apr. 2021
5	F	68	1.46×10^4	Mar. 2021
6	F	74	1.56×10^4	Dec. 2020
7	F	48	1.57×10^4	Apr. 2021
8	F	41	1.65×10^4	Dec. 2020
9	M	61	1.86×10^4	Dec. 2020
10	M	66	2.00×10^4	Apr. 2021
11	M	43	2.70×10^4	Feb. 2021
12	M	68	2.82×10^4	Apr. 2021
13	M	33	(Day 1) 5.76×10^4<br>(Day 5) 2.86×10^4	Mar. 2021

14	M	48	3.53 × 10^4	Mar. 2021
15	M	44	4.78 × 10^4	Nov. 2020
16	M	72	1.99 × 10^5	Nov. 2020
17	F	69	2.41 × 10^5	Feb. 2021

*M = male; F=female; ** = genome copies per gram of stool.

Table S3. Real-time PCR assay used for genotyping BuV-DNA positive samples.

Primers and Probe	Sequence	Position*	PCR Product Size (bp)
BuV1_Fw	GCAGACTTCCAACAACTAACAAC	315 8–3180	102[a]
BuV1_Rev	GCCTTGTTTGCTTACTGTTTTG	325 9–3280	
BuV1_Probe	GCAGAGAAGTCACACTAGAACACCTCGACC	318 6–3215	
BuV2_Fw	TGAACACATGACAGGAC	398 7–4003	124[b]
BuV2_Rev	GTATGGTCCAAATGTGTT	411 0–4127	
BuV2_Probe	CAATCAGAACTAAACGACCACATTC	403 3–4057	
BuV3_Fw	GCACAACAAACAGGMCAAACAA	399 4–4015	114[c]
BuV3_Rev	GAATGTGTTGTGGTAGTCTTGTG	410 7–4129	
BuV3_Probe	TGCACACCAATCAGAACTCAGAAATGCMACA	403 2–4062	

* = positions by reference to sequences quoted in the next column; a = NC_038544.1 Bufavirus 1 strain BF.86 NS1; b = JX027297 bufavirus-2 strain BF.39; c = NC_024888 Bufavirus-3 strain: BTN-63.
